# Digital Twin: A Future Health Challenge in Prevention, Early Diagnosis and Personalisation of Medical Care in Paediatrics

**DOI:** 10.3390/ijerph20032181

**Published:** 2023-01-25

**Authors:** Valeria Calcaterra, Valter Pagani, Gianvincenzo Zuccotti

**Affiliations:** 1 Department of Internal Medicine and Therapeutics, University of Pavia, 27100 Pavia, Italy; 2 Pediatric Department, Buzzi Children’s Hospital, 20154 Milano, Italy; 3 Grant & Research Department-LJA-2021, Asomi College of Sciences, 2080 Marsa, Malta; 4 Department of Biomedical and Clinical Science, University of Milano, 20157 Milano, Italy

Modern medicine must move from a wait-and-see and remedial system to a preventive and interdisciplinary science that aims to provide patients with personalised and precise treatment planning [1].

Personalised medicine (PM) is becoming increasingly important in the clinical and research settings of all medical disciplines, including pediatrics. PM focuses on the phenotyping of individual patients at the same clinic, allowing tailored screening, diagnostics, and treatment [2,3]. Children require particular attention because of their specificity in growth, physiology, and psychosocial development [4].

To achieve and implement the PM approach, the digital twin system (DTS) has been proposed. The DTS is composed of a physical element (the patient), a cybernetic element (the patient’s DT), and two-way interactions between the two elements. The sensors transform the signals of the patient into the DT of the patient. Artificial intelligence software processes the signals to act through recommendations or automatic adaptations of the patient’s management [5].

Due to the high complexity of the human body and its functional mechanisms (not fully elucidated), no DT of the whole human body has actually been designed [5]. However, DTS of single organs have gradually been introduced. The first implantable cardioverter-defibrillators were proposed in the 1980s. These tools detect an irregular heartbeat and automatically deliver an electric shock to restore a normal rhythm on the basis of if-else algorithms [5,6].

An artificial pancreas for children with type 1 diabetes, combining a closed-loop glucose control system and insulin infusion algorithm, opened the way for DTS in the management of paediatric chronic diseases [5].

In the near future, DTS are likely to be developed for other complex chronic diseases of paediatric age.

A future application may be for paediatric obesity, one of the most critical public health challenges. Childhood obesity is a multisystem condition that has various complications [6]. Genetic and non-genetic factors, and pre- and postnatal events, have been considered in its pathogenesis [6]. Thanks to the DTS, it will be possible to predict the risk of obesity and to monitor related complications prior to observing the symptoms. Predicting the risk of developing diseases could early offer targeted prevention and personalized care, improving outcomes and reducing healthcare costs.

This information, combined with longitudinal metabolomic, immunological, biochemical, behavioural, and gut microbiota parameters, could define a digital replica of oneself used to implement a personalized nutritional program, offering a revolution in obesity management [7].

In childhood asthma, likewise, where different determinants of asthma symptoms need to be considered, including the treatment and the environment (pollutants, allergens, weather), DTS could be used to define the appropriate treatment in real time and/or to adopt the appropriate mitigation measures in subjects at high risk of asthma, and to determine an optimal medication dose and treatment plan, leading to a decrease in both the costs and the difficulties involved in clinically managing the disease [1,8]. Associated tools, such as home spirometers, connected inhalers, air quality trackers, smartwatches, and machine learning techniques also support a DTS being developed for asthma [1,8].

The role of DTS in the management and treatment of other non-communicable diseases, such as preventable cancers, neurodegenerative disorders [9], and rare diseases, could be also monitored during childhood.

An interdisciplinary approach is mandatory for DTS proposals, as artificial intelligence, data science, and engineering concepts can identify risk factors over the course of the patient’s life, potentially enabling personalized simulation of life-course multimorbidity risk and thus improving health outcomes [10,11].

The first stage of the DTS development for healthcare is the training of a predictive model, using machine learning, by identifying key factors over the patient’s lifespan that predict the risk of later multimorbidity associated with disease [11]. Through health technology, history, demographics, lifestyle data over time of an individual physical marker, and vital signs collected by health bracelets and watches, instrumental data and several biomarkers may be collected and used to train artificial intelligence within the scope of predicting health risks using a mathematical model [11]. The inclusion of multi-omics individual analyses may result in the identification of novel mechanisms that can understand pathogenic disease mechanisms and can potentially be exploited for personalized medicine [2].

A DTS in the health system offers a virtual disease representation, offering the possibility to test scientific hypotheses and predict the interaction of pathogenic components and their effect on children and adolescents [11].

The DTS produces highly realistic models of real systems [1,5,11]. In the case of dynamically changing systems, DTS would have a life, i.e., they would change their behaviour over time and make decisions like their real counterparts. Unlike animated avatars that can only mimic the behaviour of real systems, such as real fakes, digital twins aim to be accurate ‘digital copies’ (i.e., ‘duplicates’ of reality) that can interact with reality and their physical counterparts [11,12]. Data collection can be global but detailed down to the level of individuals and their bodies, using profiling techniques such as those used by social media. Future technologies are expanding existing personalised devices, goods, and services to the areas of decision-making, behaviour, and health [3,12].

The complexity and costs of DTS will be comparable to those of projects such as the Human Genome Project; in addition, DTS may lead to an improvement in health and access to healthcare, performing early diagnosis of diseases, performing personalized treatment, and offering innovative research directions [3,12,13].

However, for the clinical implementation of DTS, a wide range of technical, medical, ethical, and theoretical challenges [5], particularly in pediatrics, will need to be solved. It is necessary to design a decision-making process able to guarantee child protection. The constant monitoring of DTS and its ad personam predictions represent additional forms of vulnerability [5].

The topic of DTS represents current and future health challenges in pediatrics as a tool which promises to promote and protect the health of children, maximize the efficiency and efficacy of PM in the healthcare system, and to offer an innovative perspective for research.
